# Hidradenoma papilliferum of the hymen: a case report

**DOI:** 10.1186/s13256-021-02786-6

**Published:** 2021-04-13

**Authors:** Ozer Birge, Mehmet Sait Bakır, Ceyda Karadag, Zivar Eldarova, Tayup Simsek

**Affiliations:** 1 Department of Gynaecology and Obstetrics, Nyala Sudan Turkey Training and Research Hospital, Nyala, Darfur Sudan; 2 Department of Gynaecology and Obstetrics, Akdeniz University Hospital, Antalya, Turkey; 3 Division of Gynecologic Oncology, Department of Gynecology Obstetrics, Akdeiz University, Antalya, Turkey

**Keywords:** Hidradenoma papilliferum, Dyspareunia, Hymen, Anogenital

## Abstract

**Background:**

Hidradenoma papilliferum is a rare benign neoplasm arising from apocrine glands. It occurs commonly on the anogenital region of middle-aged women. It usually presents as a slow growing, solitary asymptomatic, skin colored or red nodule less than 1 cm in diameter.

**Case presentation:**

The case is a 38-year-old, white woman who presented with a painful nodule occurring within a month in the himenal region of the posterior vaginal introitus. The nodule was excisied and the histology revealed a hidradenoma papilliferum. The diagnosis and treatment of hidradenoma papilliferum is possible with surgical removal and histopathological evaluation of nodules.

**Conclusion:**

When an adult woman presents with a noduler lesion in the anogenital area, sexually transmitted diseases and other benign and malignant vulvar lesions, as well as malignant transformation is very rare but,should be kept in mind; however because it has been reported and long-term clinical follow-up is suggested

## Background

Hidradenoma papilliferum is a rare, benign apocrine tumor that occurs almost exclusively in the anogenital region of midle-aged women; they are generally solid, asymptomatic, well-confined, skin colored or red, with a noduler appearance ranging in size from 0.5 cm to 1 cm [1–4]. These nodular lesions, which can be seen mostly in the vulvar and perianal genital areas, less frequently in all extragenital areas; it is more common in women of Caucasian origin between the ages of 25–66 [5].

Dyspareunia is a symptom of a variety of disease states which can have both organic and psychological dysfunction components. There are many ways of classifying dyspareunia; based on cause, onset, frequency or location. dyspareunia is found in etiological factors in vulva lesions.

Here we reported hidradenoma papilliferum of the Hymen. When the literature was investigated, we found that this case is the first case of hydradenoma papilliferum in the hymenal region; therefore we aimed to discuss and present our case in the light of the literature.

## Case presentation

**Fig. 1:**
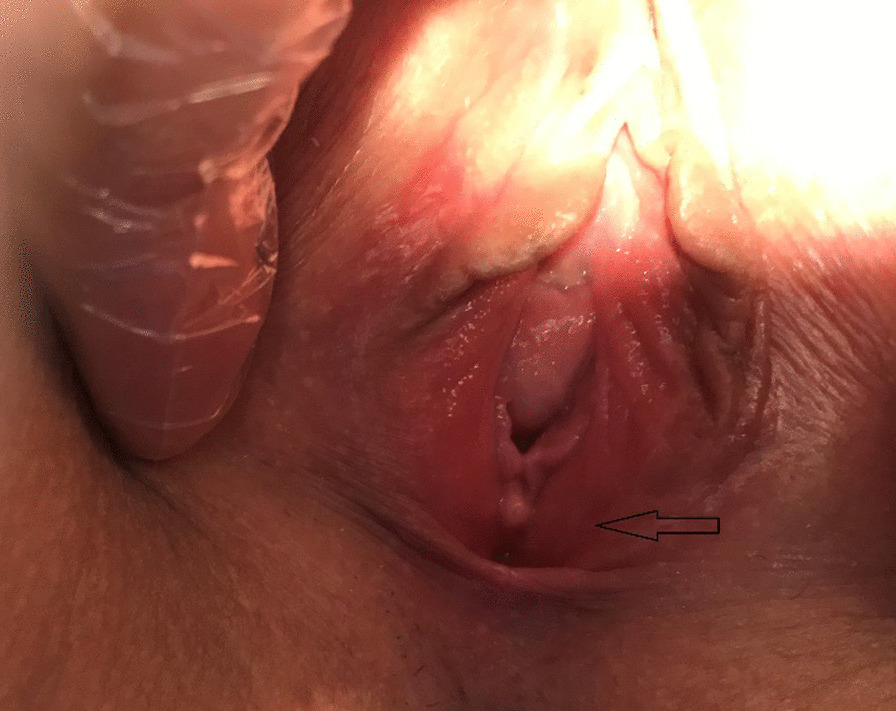
Hidradenoma papilliferum noduler lesion on posterior hymenal area.

**Fig. 2:**
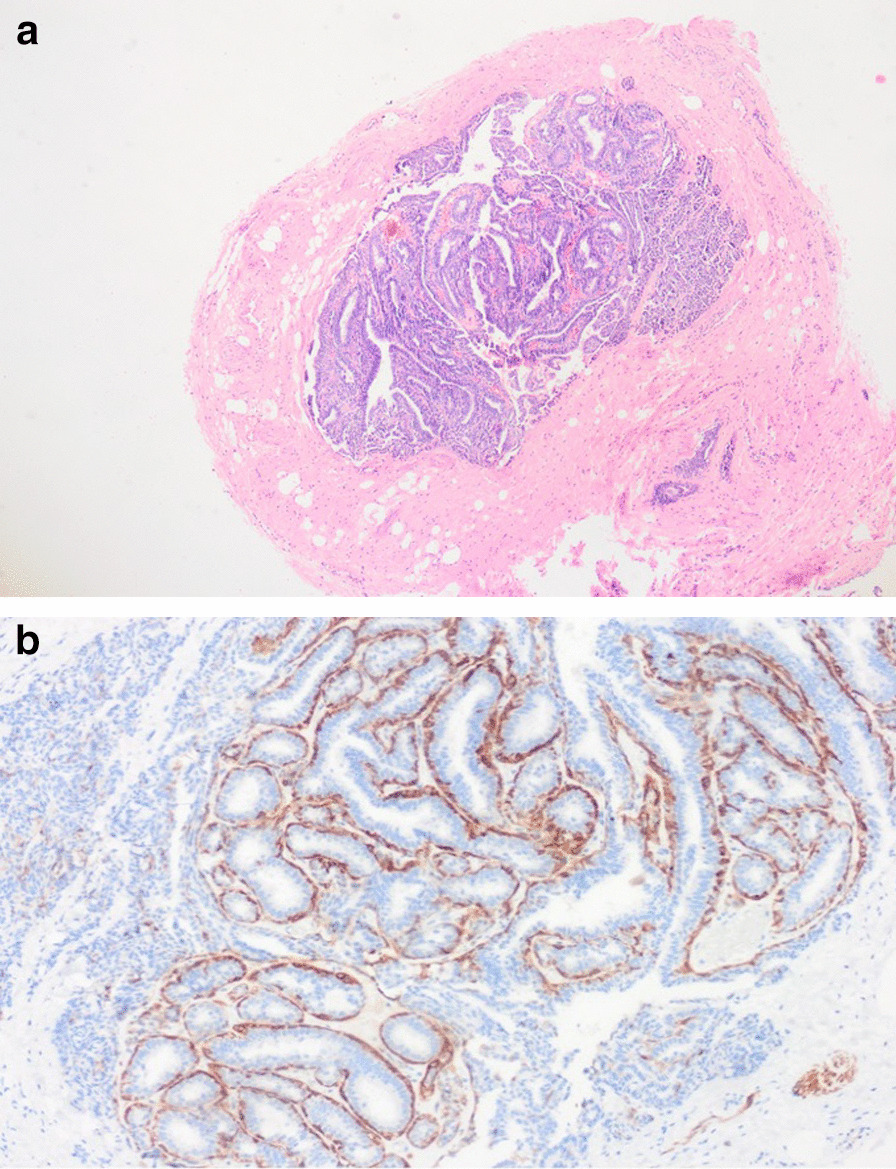
**a** Histopatological showing tumor consisted of irregular glandular structures arranged in a labyrinth pattern hidradenoma papilliferum (Hematoxylin and eosin [H&E] × 40). **b** Muscle-specific actin immunohistochemical expression in myoepithelial cells scattered among the ductal cells (× 20).

A 38 years old white woman with gravida 0 and no additional disease applied to our clinic for vulvar pain that has been going on for about 1 month. The patient did not have any additional disease and did not have any medication. She stated that she had pain in particular during sexual intercourse and her pain persisted for a few more hours after sexual intercourse. She also stated that she had a hard structure at the entrance of the vagina. In the gynecological examination of the case; hymen at 6 o'clock, nodular, approximately 1 × 2 cm, with regular borders, painful solid lesion on palpation (Figure 1). Other examination findings were normal. On the transvaginal ultrasound, the endometrium was 10 mm and regular, and tipe 6 fibroid with a size of 42 × 36 mm was seen in the posterior of the uterus corpus. Ovaries were normal. Also it was seen that cervical cytological screening of the case was not performed. The patient underwent HPV (human papilloma virus) test and pap-smear. Cervical conization was performed in the case with positive HPV 16 and 31 types and HGSIL (high grade squamous intraepithelial lesion). The nodular lesion at 6 o'clock at hymen was totally exised during the conization process and sent for pathological examination. Histopathological examination of the patient's conization material was reported as LGSIL (low grade squamous intraepithelial lesion), and as a result of the pathological examination of the hymenal lesion, hydradenoma papilliferum (Fig. 2a, b). No pain was identified after excision during sexual intercourse. Since hidradenoma papilliferum is a benign disease, no additional treatment was required. Follow-up of the patient is still ongoing for cervical premalignant lesions.

## Discussion

Hidradenoma papilliferum was first described by Worth in 1878; it is a noduler, benign neoplasm originating from apocrine glands in middle aged women, usually 30–49 years old [5]. The most common site in the body is the anogenital region, especially the labia majora. In addition to the vulva, less frequently the tumor can be seen in the interlabial sulcus, clitoris, posterior fourchette, perineum, anal region and extragenital areas [1–5]. Rare forms called ectopic hidradenoma papilliferum have been described in areas containing modified apocrine glands such as sacred skin, neck, and female and male breast, as well as in the outer ear and eyelid [6]. Although most cases are asymptomatic, single, solitary nodule, cases with mild tenderness due to perianal region have also been reported [7, 8]. Benign lesions to be considered in its differential diagnosis are hemorrhoidal disease, anorectal abscess, viral verrucous lesion, sebaceous cyst, mucous cysts, lipoma and neurofibroma, and few malignant lesions such as metastatic papillary carcinoma, syringocystoadenocarcinoma papilliferum and kuamoz cell cancer (SCC) should also be ruled out before its diagnosis [9]. Although dyspareunia is not uncommon, the reason is not clear. however, its importance should not be forgotten, although it is a rare cause in lesions of the vulva.

Today, most authors state that these lesions originate from breast-like apocrine glands [10]. In histopathological examination, it is defined as lesions, which are independent of the epidermis layer of the skin and are covered with secretory cells containing tubular structure, containing cystic and papillary structures, and also containing breast-like accessory glands [10, 11]. Several studies have identified HPV types 16, 31, 33, 53 and 56 DNA in hidradenoma papilliferum tissue. However, in these studies, it has not been proven that HPV plays role in hidradenoma papilliferum etiology [11]. Our case was 38 years old, and her routine gynecological follow-up and cervical cytological screening were not performed. In the HPV test of the case, high risk HPV types such as 16, 31 were positive, also conization was performed to the patient who had HGSIL as result of cervical cytology. A good assessment should also be made in terms of sexually transmitted diseases, since most of the cases detected hidradenoma papilliferum are of reproductive age and are sexually active. In addition, they should be screened for benign and malign tumoral lesions that may develop in the genital or extragenital areas; and if necessary, their treatment should be planned [12].

Since our case was sexually active, we performed the necessary examinations and planned the treatment for HPV and other sexually transmitted diseases. When the literature is investigated, in a study in which 14 vulvar and perianal hydroadenoma papilliferum cases with the highest number of cases were analyzed retrospectively; It was determined that the average age of the patients was 48, most of them had asymptomatic and slowly growing nodular lesions of 1 cm, and 15% of these lesions were seen as ulcers. Simple excision was performed to all cases; only one case was observed recurrence in the 6th year after excision [13]. Another article stated that a 5-mm vulvar nodular lesion detected in a 39-week asymptomatic pregnant hydradenoma papilliferum case was excised following cesarean delivery and increased lactational hormones may play a role in etiology during pregnancy [14]. BRCA1, BRCA2 and PIK3CA mutations were investigated in terms of benign and malign differentiation in anogenital and breast gland-like lesions by Konstantinova *et al.* No mutation was detected in these lesions, but they found that the PI3K-AKT pathway was active [15].

Although malignant transfarmation is very rare, there are two reported invasive cases (malignant perianal papillary hidradenoma and vulvar adenosquamous carcinoma) [10]. Local excision with a margin will be sufficient for its diagnosis, treatment, and cure [9–11]. In our case, simple total excision was made and follow-up was recommended because the pathology result was evaluated as benign.

## Conclusion

When an adult woman presents with a noduler lesion in the anogenital area, sexually transmitted diseases and other benign and malignant vulvar lesions, as well as Hidradenoma papilliferum should be kept in mind. Patient history and clinical findings are not specific for hidradenoma papilliferum and surgical removal of the lesion and its histopathological evaluation are required to make a definitive diagnosis.

## Data Availability

The authors agree to make the raw data and materials described in our manuscript freely available.
